# Comparison Between Non-Enhanced Magnetic Resonance Angiography (MRA) and Digital Subtraction Angiography (DSA) for the Detection of Intratumoral Aneurysms in Renal Angiomyolipoma (Renal AML)

**DOI:** 10.3390/jcm14155276

**Published:** 2025-07-25

**Authors:** Daisuke Yashiro, Yoshiki Kuwatsuru, Hiroshi Toei, Takeshi Udagawa, Shingo Okada, Hitomi Kato, Naoko Saito, Ryohei Kuwatsuru

**Affiliations:** 1 Department of Radiology, Graduate School of Medicine, Juntendo University, 2-1-1 Hongo, Bunkyo-ku, Tokyo 113-8421, Japany-kuwatsuru@juntendo.ac.jp (Y.K.); h-toei@juntendo.ac.jp (H.T.); t-udagawa@juntendo.ac.jp (T.U.); n.saito.du@juntendo.ac.jp (N.S.); 2 Center for Promotion of Data Science, Graduate School of Medicine, Juntendo University, 2-1-1 Hongo, Bunkyo-ku, Tokyo 113-8421, Japan

**Keywords:** flow-in balanced SSFP, non-enhanced MRA, renal AML

## Abstract

**Background/Objectives:** To evaluate the diagnostic performance of non-enhanced MRA in detecting intratumoral aneurysms in renal AML, using digital subtraction angiography (DSA) as the reference standard. **Methods:** Fourteen female patients (mean age, 39 years; range, 21–57 years) who received prophylactic transcatheter arterial embolization (TAE) for non-hemorrhagic renal AML(s) between July 2010 and September 2018 were included in this study. All received a non-enhanced MRA scan prior to TAE. Non-enhanced MRA images were obtained using the flow-in technique with three-dimensional balanced steady-state free precession (SSFP). The MRA and DSA images were jointly evaluated by three radiologists. In this study, significant aneurysms were defined as aneurysms with a diameter of 3 mm or more within the renal AML. The MRA images assessed the number and location of significant aneurysms. The DSA images were used as the reference standard. **Results:** DSA identified 30 significant aneurysms in eight kidneys; MRA identified 26, giving a sensitivity of 87%. There were no false positives, resulting in a specificity of 100%. **Conclusions:** Flow-balanced SSFP MRA is effective in detecting significant aneurysms in renal AML and could be a viable alternative for patient follow-up.

## 1. Introduction

Renal angiomyolipoma (AML) is one of the most common solid benign renal tumors composed of fat, smooth muscle, and blood vessels. There are two main types of renal AML, one associated with tuberous sclerosis complex (TSC) (20%) and the other sporadic (80%) [1].

The vessels of renal AMLs are fragile and often rupture, which can be fatal. Therefore, renal AML requires careful follow-up, and prophylactic tumor embolization may be performed, especially in cases where the total AML diameter is more than 4 cm or the AML contains an aneurysm more than 5 mm in diameter, both of which are considered rupture-prone [2,3]. Contrast-enhanced computed tomography angiography (CTA) or magnetic resonance angiography (MRA) is used in the follow-up of renal AMLs. While contrast studies allow for more accurate assessment of aneurysms, they also require attention to allergic side effects and decreased renal function [4,5]. In addition, due to the risk of nephrogenic systemic fibrosis (NSF), MRA with contrast agents may not be possible in patients with impaired renal function [6].

In recent years, non-contrast-enhanced (NCE) MRA, which does not use contrast agents, has attracted attention as a method for evaluating blood vessels in the abdominal region [7,8]. In particular, the technique of NCE MRA based on balanced steady-state free precession (SSFP, or flow-in balanced SSFP) has shown great promise in the evaluation of renal artery stenosis [7,8,9,10]. Similarly, if flow-in balanced SSFP can be used to evaluate renal AML aneurysms, it would benefit many AML patients by allowing follow-up without using contrast agents, which carry the risk of side effects and reduced renal function.

Although it is said that aneurysms in AML with a diameter of 5 mm or more are prone to rupture, there are cases in which aneurysms with a diameter of 3 mm are observed and grow rapidly, so this study aims to evaluate the diagnostic performance of flow-in balanced SSFP in detecting aneurysms larger than 3 mm in renal AML using digital subtraction angiography (DSA) as the reference standard.

## 2. Materials and Methods

### 2.1. Subjects

The institutional review board approved this research, and because of the retrospective nature of this study, the requirement of informed consent was waived. From July 2010 to September 2018, fourteen consecutive female patients (mean age, 39 years; range of age, 21–57 years) who received prophylactic transcatheter arterial embolization (TAE) for non-hemorrhagic renal AML(s) in our department were included in this study. Also, no cases were excluded based on tumor size, site of presence, gender, or previous medical history. All patients underwent NCE MRA before TAE. The interval between MRA and TAE was not more than 90 days (mean days, 37; range of days, 1–88). Nine patients had bilateral tumors, and five patients had unilateral tumors. Therefore, a total of 23 AMLs had available MRA and DSA images except for the multiple small AMLs seen in tuberous sclerosis and lymphangioleiomyomatosis.

### 2.2. Imaging Protocol

All examinations were conducted in a 1.5-T MR unit, using a pair of phased-array coils (16 channels with 16 elements: Atlas Speeder Body Coil combined with Atlas Speeder Spine Coil; Toshiba Medical Systems, Otawara, Japan) placed at the front and back of the abdomen. Our imaging protocol for patients includes coronal and axial T2-weighted imaging (T2-Cor, T2-Ax), axial T2-weighted imaging with fat saturation (T2 FatSup-Ax), heavy T2-weighted axial imaging (HeavyT2-Ax), T1-weighted axial imaging using in-phase and out-phase dual echo (T1 [in/out dual echo]-Ax), 3D T1-weighted imaging with fat saturation (3DT1 FatSup), diffusion-weighted axial imaging (DWI-Ax [b-value = 1000]), and unenhanced 3D True-SSFP time-SLIP imaging in both coronal and axial planes (3DTrue-SSFP time-Slip [Cor, Ax]).

The imaging technique for 3D True-SSFP time-SLIP imaging was the same as in previous reports evaluating renal artery delineation with SSFP [11]. In this method, a fixed BBTI value (1500 ms) was used, and neither navigator nor respiratory gating was applied, allowing for a reproducible imaging technique with minimal equipment dependency, which facilitates clinical implementation. For this reason, the imaging approach used in the referenced study was adopted in the present research. Although SSFP is known to be sensitive to motion, pre-scan breathing training was provided to stabilize the patient’s respiration, and a relatively rapid acquisition protocol was employed to minimize respiratory effects. Moreover, in AML patients, the kidneys typically contain relatively large tumors, which are expected to reduce respiratory-induced kidney motion compared to normal kidneys. Abdominal compression was not used in this study, considering the potential risk of triggering aneurysm rupture.

### 2.3. DSA Technique

All procedures were performed by the same senior interventional radiologist (R.K., who has more than 10 years of experience in performing TAE of renal AML). A 4-French (Fr) vascular sheath (Medikit Supersheath; Medikit Co., Ltd., Tokyo, Japan) was placed in the right common femoral artery under local anesthesia. The tip of a 4-Fr catheter was placed in the right or left renal artery trunk using an angled guide wire (Radiofocus Guide Wire M; Terumo Co., Ltd., Tokyo, Japan), and initial renal angiography was performed at a flow rate of 3 mL/sec of iodinated contrast material (iopromide 300 [Ultravist^®^; Bayer AG, Leverkusen, Germany]) with total amount of 12 mL using a digital subtraction angiography unit (INFX-8000C/JL; Canon Medical Systems, Tokyo, Japan) to evaluate the renal artery, tumor stain, aneurysms, and detect possible additional tumor feeder vessels. For the DSA imaging, we used the Auto exposure control system. From this point, superselective catheterization to the tumor vessels was performed using a micro-catheter over a micro-guidewire. The territory supplied by the target vessel was confirmed, and embolic material was administered to the site.

### 2.4. Image Analysis

In this study, three radiologists (each reader had >5 years’ experience) assessed whether flow-balanced SSFP could confirm an aneurysm. Significant aneurysm size was defined as 3 mm or greater, considering the indications for prophylactic IVR treatment in AML.

In the evaluation, the radiologists reviewed the MIP image and the original MRA image and determined the location and number of aneurysms. The DSA images were then reviewed to confirm the location and number of aneurysms. If the opinions of each radiologist differed, they were all reviewed together to determine the final location and number of aneurysms. Confirmation of the existence of the aneurysm was performed by three radiologists who reviewed the angiography images to reach a consensus.

### 2.5. Statistical Analysis

Sensitivity, specificity, false positive rate, and false negative rate of MRA in detecting significant aneurysms were determined, using the DSA as the reference standard. This study evaluated only the presence or absence of aneurysms on MRA and DSA and did not include analysis of bias using Bland–Altman plots.

### 2.6. Generative AI

In this study, generative AI (GPT-4o, May 2024 release) was used for English editing, refinement of expressions, and literature search during manuscript preparation. The generative AI was not used for study design, data analysis, or interpretation.

## 3. Results

MRA image quality was maintained to the extent that even the second branch of the renal artery was visible on all images. In total, DSA identified 30 significant aneurysms in eight kidneys—14 in three right kidneys and 16 in five left kidneys. MRA successfully detected 26 of these 30 aneurysms, resulting in a sensitivity of 87% (26/30). Importantly, MRA demonstrated specificity, as no significant aneurysms were found in the 11 kidneys that were confirmed aneurysm-free by DSA, resulting in a false positive rate of 0% (Table 1).

**Figure 1:**
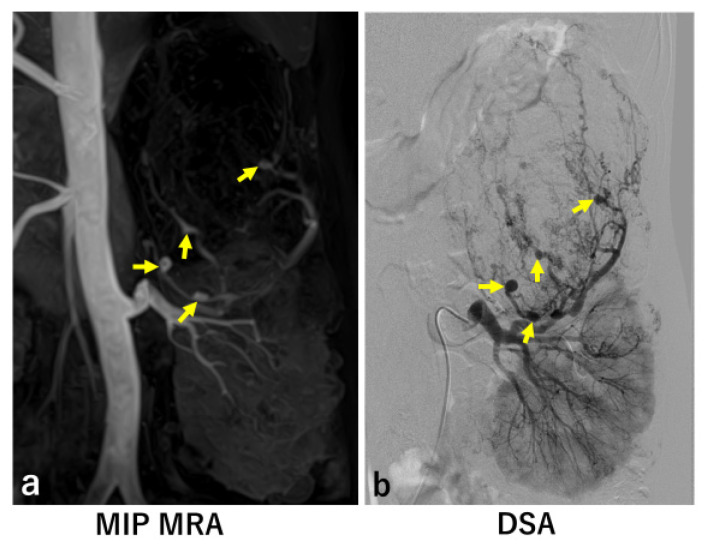
Case 5. MIP MRA (**a**) showed four significant intratumoral aneurysms (arrows). Each was confirmed by renal DSA (**b**).

**Figure 2:**
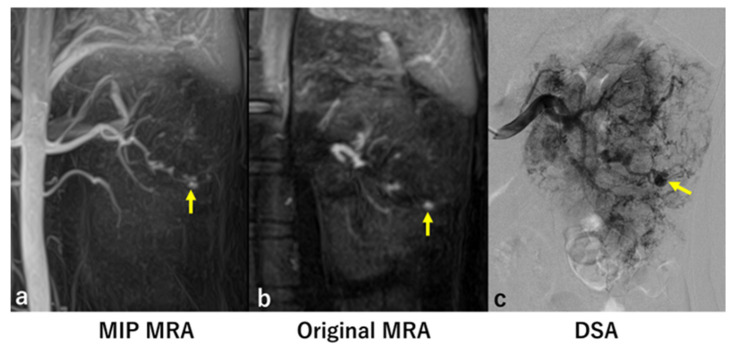
Case 11. Both MIP MRA (**a**) and original MRA (**b**) showed an intratumoral aneurysm with a diameter of 5 mm (arrow). DSA (**c**) before embolization showed the 5 mm intratumoral aneurysm (arrow).

**Figure 3:**
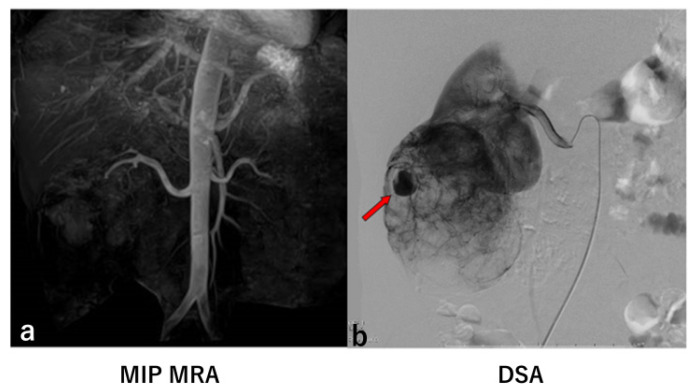
Case 7. MIP MRA (**a**) showed no significant aneurysm. Renal DSA (**b**) showed an intratumoral aneurysm with a diameter of 14 mm (arrow). MRA showed only proximal part of bilateral renal arteries.

**Figure 4:**
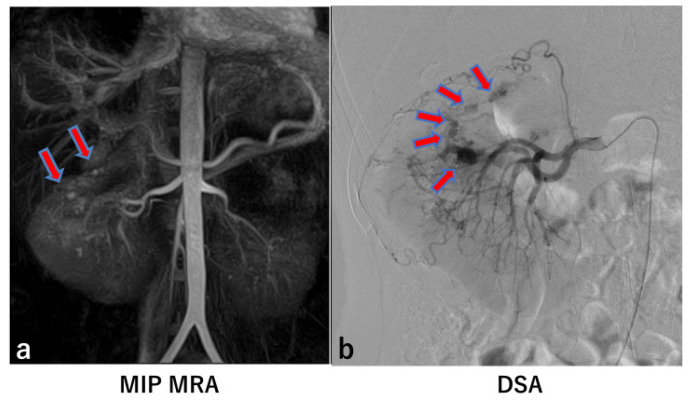
Case 13. Two significant aneurysms were detected on MIP MRA (arrows) (**a**). On the other hand, renal DSA showed five significant aneurysms (arrows) (**b**).

In six of the eight kidneys with significant aneurysms identified by DSA, MRA detected the same number of aneurysms as DSA (Figure 1 and Figure 2). However, in two kidneys, MRA missed a total of four significant aneurysms (Figure 3 and Figure 4).

### Introduction to Two False Negative Cases

In this study, four significant aneurysms were not detected by MRA (Figure 2 and Figure 4). In Figure 4 (Case 13), two significant aneurysms were identified on maximum intensity projection (MIP) MRA; however, digital subtraction angiography (DSA) revealed the presence of five significant aneurysms. The three aneurysms missed on MIP MRA were also not visible on the source images. Notably, in Case 13, the visualization of the peripheral renal arteries on MRA was generally poor.

One potential explanation for this finding may be related to the flow-in balanced SSFP sequence used in this study. The blood-bolus tagging interval (BBTI) in this sequence is designed to correspond to the distance and time required for blood to travel to the renal arteries [11]. In this case, the patient’s blood flow velocity may have been slower than anticipated, leading to suboptimal BBTI selection. To address this issue, the use of a 2D BBTI preparatory scan, which generates images at various BBTI settings before the 3D acquisition, could assist in determining the most appropriate BBTI for individual patients.

In addition to flow-related factors, respiratory motion may have contributed to the unclear imaging in Case 13. Suboptimal respiratory synchronization is a common problem in MRA, especially when a patient’s breathing pattern is irregular. In such cases, the resulting images tend to be blurred [12]. To mitigate this problem, it is important to instruct patients to maintain a consistent breathing pattern throughout the scan. In some cases, such as obese patients, the use of an abdominal compression belt may be beneficial to stabilize the diaphragm and reduce motion artifacts [12].

In Case 7 (Figure 3), no significant aneurysm was detected on MIP MRA, whereas DSA revealed an intratumoral aneurysm measuring 14 mm in diameter. Upon retrospective review, it was determined that the aneurysm visualized on DSA was located outside the MRA imaging range. This discrepancy occurred because the flow rate of AML was slow, or the aneurysm was located outside the MRA scan range.

## 4. Discussion

In this study, we evaluated the diagnostic performance of NCE MRA using SSFP in the detection of intratumoral renal aneurysms in patients with AML. The sensitivity of SSFP-MRA for the detection of renal aneurysms was 87% (26/30), with no false-positive results (0%). In six of eight kidneys with aneurysms confirmed by DSA, the presence and number of aneurysms detected by MRA were consistent with those identified by DSA, resulting in a sensitivity of 75%. Importantly, MRA did not misidentify aneurysms in the 11 kidneys confirmed to be aneurysm-free by DSA, demonstrating a specificity of 100% and a negative predictive value of 85%. These results underscore the high specificity of MRA with SSFP in detecting significant aneurysms in renal AML patients.

The clinical significance of these results is particularly relevant because small intratumoral aneurysms rarely rupture [13,14]; however, renal AMLs, especially the cases in which the diameter of intratumoral aneurysms are more than 5 mm in diameter, are prone to spontaneous rupture [2,3], which can lead to life-threatening hemorrhage. Therefore, careful and regular imaging follow-up is essential to monitor changes in aneurysm size and morphology. Current practice often involves the use of contrast-enhanced CT or MRI for this purpose. However, the administration of contrast agents, whether for CT or MRI, carries inherent risks, including contrast-induced nephropathy and allergic reactions [4,5]. In particular, gadolinium-based contrast agents used in MRI are contraindicated in patients with severely impaired renal function due to the risk of NSF [6]. This limitation highlights the need for alternative imaging modalities in certain patient populations.

Our findings contribute to the emerging body of evidence that suggests NCE MRA with SSFP may offer a reliable, non-invasive option for the follow-up of patients with renal AML, particularly in those who are at risk for complications associated with contrast agents. Further studies are warranted to validate these results in larger cohorts and to explore the potential of NCE MRA in other vascular abnormalities associated with AML.

Recent advances in non-contrast-enhanced MRA (NCE-MRA) techniques have further expanded the clinical utility of contrast-free vascular imaging, particularly for abdominal and renal applications. Among these, quiescent-interval slice-selective (QISS) MRA has demonstrated high diagnostic accuracy in peripheral and renal arteries, supported by optimized ECG gating and radial k-space sampling [15,16]. Similarly, arterial spin labeling (ASL) has been increasingly used to assess renal perfusion without the need for contrast agents, allowing for quantitative evaluation of blood flow [17]. In addition, 4D flow MRI offers comprehensive hemodynamic information, enabling visualization of flow patterns, velocity, and wall shear stress in the renal vasculature [18,19].

Several recent studies have highlighted the feasibility and diagnostic performance of these techniques in comparison with contrast-enhanced MRA or CTA [20,21]. These developments underscore the growing role of non-contrast MRA in vascular imaging. In this context, our study supports the clinical applicability of flow-in balanced SSFP as a simpler, yet highly specific alternative, especially in patients with contraindications to contrast agents.

Several limitations of our study should be acknowledged. First and foremost, the retrospective nature of our analysis, combined with the small sample size, limits the generalizability of our findings. The rarity of AML, along with the selection criteria requiring that all patients undergo the complete set of examinations on the same 1.5-T MR unit, further constrained the size of our cohort. In addition, NCE MRA may have limitations in detecting aneurysms in areas of reduced perfusion or slow flow, which may underestimate the true burden of aneurysms in certain patients [11]. Therefore, future research should focus on comparing NCE MRA with contrast-enhanced MRA and conventional angiography to better understand the strengths and limitations of each modality in this clinical context.

Advances in imaging technology, coupled with increasing concerns about the safety of gadolinium-based contrast agents, including issues related to gadolinium retention in the brain and the risk of NSF in patients with chronic kidney disease, have spurred interest in the development of non-contrast imaging techniques [6]. The results of our study suggest that NCE MRA with SSFP could play a significant role in the future of AML management, particularly in patients where the use of contrast agents is either not feasible or contraindicated.

We hope that our findings will serve as a foundation for future investigations and inspire further studies that explore the utility of non-contrast-enhanced imaging modalities in the assessment and follow-up of vascular abnormalities in patients with renal AML and other related diseases. Expanding the literature on NCE imaging will provide valuable insights that could ultimately refine clinical practice and improve patient outcomes.

## 5. Conclusions

Flow-in balanced SSFP may be an appropriate non-enhanced MR angiographic technique for the detection of ≥3 mm aneurysms within renal AML and may be a potential alternative for the follow-up of renal AML patients who are unable to undergo CTA or contrast-enhanced MRA.

## Figures and Tables

**Table 1:** Comparison of aneurysms detected by MRA and DSA.

No. of Cases	Age /Sex	Concurrent Disease	AML Side	Number and Size of Aneurysms (Right)	Number and Size of Aneurysms (Left)	Maximum Size of Angiomyolipoma
				MRA/DSA	MRA/DSA	
1	57/F	TSC	Right	0/0	N/A *^1^	Multiple *^2^ (153 × 113 × 59 mm)
2	29/F	TSC	Bilateral	0/0	1/1 (3 × 2 mm/4 × 2 mm)	Multiple *^2^ (142 × 119 × 76 mm)
3	38/F	sLAM	Left	N/A *^1^	0/0	154 × 91 × 71 mm
4	37/F	sLAM	Left	N/A *^1^	7/7 (max. 9 × 6 mm, min. 4 × 3 mm /max. 9 × 7 mm, min. 4 × 3 mm)	110 × 86 × 86 mm
5	37/F	TSC	Bilateral	0/0	4/4 (max. 6 × 2 mm, min. 3 × 2 mm /max. 6 × 4 mm, min. 4 × 3 mm)	Multiple *^2^ (130 × 125 × 85 mm)
6	21/F	TSC	Bilateral	0/0	0/0	Multiple *^2^ (78 × 74 × 67 mm)
7	50/F	sAML	Right	0/1 (14 × 13 mm)	N/A *^1^	105 × 91 × 77 mm
8	55/F	TSC	Bilateral	8/8 (max. 10 × 7 mm, min.3 × 2 mm /max. 12 × 6 mm, min. 4 × 3 mm)	N/A *^1^	Multiple *^2^ (119 × 102 × 83 mm)
9	33/F	TSC	Bilateral	0/0	3/3 (max. 5 × 3 mm, min. 3 × 2 mm /max. 4 × 3 mm, min. 3 × 3 mm)	Multiple *^2^ (115 × 109 × 67 mm)
10	33/F	sLAM	Bilateral	0/0	N/A *^1^	Multiple *^2^ (121 × 84 × 63 mm)
11	33/F	TSC	Bilateral	N/A *^1^	1/1 (5 × 3 mm/4 × 3 mm)	Multiple *^2^ (76 × 59 × 48 mm)
12	30/F	TSC	Bilateral	0/0	N/A *^1^	Multiple *^2^ (54 × 50 × 32 mm)
13	33/F	sLAM	Bilateral	2/5 (max. 6 × 4 mm, min. 4 × 3 mm /max. 8 × 6 mm, min. 3 × 3 mm)	0/0	Multiple *^2^ (85 × 83 × 75 mm)
14	37/F	sAML	Left	N/A *^1^	0/0	42 × 39 × 30 mm

*^1^ N/A; not applicable. *^2^ Multiple; more than one angiomyolipomas detected.

## Data Availability

All the data (MRA, DSA, and attributes of the patients) in this study are obtained in our institute, and available from the corresponding author (R.K.) upon reasonable request.
